# *Mycobacterium avium* subsp. *paratuberculosis* antigens induce cellular immune responses in cattle without causing reactivity to tuberculin in the tuberculosis skin test

**DOI:** 10.3389/fimmu.2022.1087015

**Published:** 2023-01-18

**Authors:** Sandeep K. Gupta, Tania Wilson, Paul H. Maclean, Bernd H. A. Rehm, Axel Heiser, Bryce M. Buddle, D. Neil Wedlock

**Affiliations:** ^1^ AgResearch, Hopkirk Research Institute, Palmerston North, New Zealand; ^2^ AgResearch, Grasslands, Palmerston North, New Zealand; ^3^ Centre for Cell Factories and Biopolymers, Griffith Institute for Drug Discovery, Griffith University, Brisbane, QLD, Australia; ^4^ Menzies Health Institute Queensland (MHIQ), Griffith University, Gold Coast, QLD, Australia

**Keywords:** *Mycobacterium avium* subspecies *paratuberculosis*, Johne’s disease, recombinant MAP fusion protein particle vaccine, IFN-γ, IL-17, nanostring, gene expression, tuberculin skin test

## Abstract

*Mycobacterium avium* subspecies *paratuberculosis* (MAP) causes chronic progressive granulomatous enteritis leading to diarrhea, weight-loss, and eventual death in ruminants. Commercially available vaccine provides only partial protection against MAP infection and can interfere with the use of current diagnostic tests for bovine tuberculosis in cattle. Here, we characterized immune responses in calves to vaccines containing four truncated MAP antigens as a fusion (Ag85A^202-347^-SOD^1-72^-Ag85B^173-330^-74F^1-148+669-786^), either displayed on protein particles, or expressed as a soluble recombinant MAP (rMAP) fusion protein as well as to commercially available Silirum^®^ vaccine. The rMAP fusion protein elicited the strongest antigen-specific antibody responses to both PPDA and recombinant antigen and strong and long-lasting T-cell immune responses to these antigens, as indicated by increased production of IFN-γ and IL-17A in antigen-stimulated whole blood cultures. The MAP fusion protein particle vaccine induced minimal antibody responses and weak IFN-γ responses but stimulated IL-17A responses to recombinant antigen. The immune response profile of Silirum^®^ vaccine was characterized by weak antibodies and strong IFN-γ and IL-17A responses to PPDA. Transcription analysis on antigen-stimulated leukocytes from cattle vaccinated with rMAP fusion protein showed differential expression of several immune response genes and genes involved in costimulatory signaling, *TLR4*, *TLR2*, *PTX3*, *PTGS2*, *PD-L1*, *IL1B*, *IL2*, *IL6*, *IL12B*, *IL17A*, *IL22*, *IFNG*, *CD40*, and *CD86*. Moreover, the expression of several genes of immune pathways correlated with cellular immune responses in the rMAP fusion protein vaccinated group. These genes have key roles in pathways of mycobacterial immunity, including autophagy, manipulation of macrophage-mediated killing, Th17- and regulatory T cells- (Treg) mediated responses. Calves vaccinated with either the rMAP fusion protein or MAP fusion protein particle vaccine did not induce reactivity to PPDA and PPDB in a comparative cervical skin test, whereas Silirum^®^ induced reactivity to these tuberculins in most of the vaccinated animals. Overall, our results suggest that a combination of recombinant MAP antigens in the form of a soluble fusion protein vaccine are capable of inducing strong antigen-specific humoral and a balanced Th1/Th17-cell immune response. These findings, together with the absence of reactivity to tuberculin, suggest this subunit vaccine could provide protective immunity against intracellular MAP infection in cattle without compromising the use of current bovine tuberculosis surveillance test.

## Introduction

Johne’s disease (JD) or paratuberculosis is caused by *Mycobacterium avium* subspecies *paratuberculosis* (MAP), and results in chronic progressive granulomatous enteritis affecting ruminants (1, 2). Animals with clinical infection are often culled due to chronic diarrhea, gradual weight loss, and reduced milk production, resulting in considerable economic losses to the livestock industry worldwide (3, 4). The only commercially available vaccine for cattle, Silirum^®^ (Zoetis, NSW, Australia), provides partial protection by reducing bacterial shedding in feces and the severity of JD (5). The vaccine is comprised of heat-killed MAP and interferes with the use of the tuberculin skin test for bovine tuberculosis (6, 7). A more effective vaccine against MAP infection without sensitizing animals to tuberculin is required.

New approaches of developing a vaccine against MAP infection have been proposed including, protein-based subunit vaccines, DNA vaccines and live vector vaccines (8–10). However, the level of protection induced by these types of vaccines has not exceeded the levels conferred by live attenuated or killed MAP vaccines (11). Efficient and targeted delivery of antigens to antigen-presenting cells (APCs) is crucial to induce effective protective immune responses (12–14). Advances in particulate-type vaccines hold promise for improved vaccines due to their efficient uptake by APCs (12–14). A wide range of approaches are being used for enhanced antigen delivery, including formulation of antigens in particulate adjuvants, such as liposomes and microparticles as well as particles displaying antigens such as virus-like particles, bacteria-based vectors, liposomes, immune-stimulating complexes, inclusion bodies, and protein particles (15–17).

Protein particles have several advantages for vaccine antigen delivery. The large surface area of protein particles, along with the co-delivery of multiple antigens on the same particle leads to better activation of APCs (18–20). In addition, their low production cost and ease of manufacture process have made protein particles an attractive choice for use in vaccine formulation (21–23).

Studies have demonstrated that MAP antigens including antigen complex 85A (Ag85A), Ag85B, Ag85C, and superoxide dismutase (SOD) and a polyprotein 74F produced as recombinant soluble proteins in *E. coli* (24, 25) or truncated fusion secretory proteins (Ag85A^202-347^-SOD^1-72^-Ag85B^173-330^ and 74F^1-148+669-786^) in two Salmonella vectors (26) can induce protective immunity against MAP infection in mice. In a previous study, we demonstrated that protein particles displaying different regions of MAP antigens Ag85A, Ag85B, SOD and 74F as well as soluble form of these antigens induced strong antigen-specific T-cell immune responses and provided protection against MAP challenge in mice (27). In the current study in cattle, we investigated the ability of protein particles displaying Ag85A, SOD, Ag85B and 74F to induce antibody and cellular immune responses and compared them to the MAP antigens expressed as a single fusion soluble recombinant protein and the commercial vaccine Silirum^®^. We also tested the reactivity of the vaccinated animals to bovine purified protein derivatives in the intradermal tuberculin skin test.

## Materials and methods

### Animals

Thirty-two Holstein-Friesian cattle, 2-3 months old were sourced from a commercial farm with no history of JD. Prior to the trial, the cattle (n = 32) tested negative for reactivity to protein purified derivative from *Mycobacterum avium* (PPDA) (Prionics, Schlieren-Zurich, Switzerland) in the whole-blood interferon-γ (IFN-γ) assay and were selected from a larger group of animals (n = 45). The cattle were grazed on pasture in a separate paddock during the trial.

### Production and purification of protein particle and recombinant protein

Protein particles displaying MAP fusion antigen were produced as described previously (27). The coding sequence for truncated MAP fusion antigens (Ag85A^202-347^-SOD^1-72^-Ag85B^173-330^-74F^1-148+669-786^) was fused to the N-terminal of PhaC protein coding sequence in pPolyN plasmid using the strategy as described previously (27). The resultant plasmid was transformed into *E. coli* BL21 (DE3) cells (ThermoFisher Scientific, New Zealand) to produce protein particles displaying MAP fusion antigen fused to PhaC. Briefly, transformed *Escherichia coli* BL21 (DE3) cells were grown in Luria Broth supplemented with 75 µg/mL ampicillin (Sigma, St. Louis, MO) in a shaking incubator at 37°C. The cultures were induced with 0.5 mM of isopropyl β-D-1-thiogalactopyranoside (IPTG) (Sigma, St. Louis, MO) until an OD_600_ of 0.5 was reached, and the cultures were further incubated for 48 h at 25°C with shaking at 200 rpm. The cells were lysed using a microfluidizer and the lysate was centrifuged at 8,000 × g for 15 min at 4°C to purify the protein particles. The purified protein particles were treated with 70% ethanol for 1 h to kill any residual bacteria. The protein particles were washed twice in cold phosphate-buffered saline (PBS), 10 mM, pH 7.3 and re-suspended in PBS as a 20% slurry. Sterility of the protein particles was confirmed by plating an aliquot of the slurry onto LB and incubating for 2 days at 37°C.

The coding sequence for the MAP fusion antigen was cloned into the pET151 expression vector and used to produce MAP fusion protein as a recombinant protein with a 6x histidine tag in *E. coli* BL21 (DE3) cells as described previously (27). The his-tagged protein was purified using a gravity flow nickel-chelate (Ni-NTA) column (Takara Bio, CA, USA) and treated with Triton X-114 to reduce endotoxin contamination (28, 29).

### Vaccine preparation

Vaccines were prepared as previously reported (27). Briefly, vaccines were prepared by formulating either PBS alone, recombinant MAP (rMAP) fusion protein or protein particles displaying MAP fusion antigen (300 µg per vaccine dose) with Emulsigen-D (20%, vol/vol, MVP Laboratories, Omaha, NE). The concentration of MAP fusion antigen in protein particles was calculated (**Table 1** and **Figure S1**) according to a previously published method (30). Silirum^®^ vaccine containing heat-inactivated MAP strain 316F was purchased from Zoetis, NSW, Australia.

**Table 1:** Concentration of MAP fusion antigen in protein particles.

	Amount of PhaC-MAP antigen fusion/wet particles (ng protein/mg beads)	µg particles loaded	ng MAP fusion protein per µg particles	total MAP fusion protein weight MW	MAP fusion protein component MW	MW ratio fusion protein to PhaC	ng MAP fusion protein per µg particles	µg MAP fusion protein per mg particles (average)	mg of particles required for 300 µg antigen
PhaC-MAP fusion antigen	219	15	14.60	130	65.7	0.51	7.38	5.96	50.31
	96	7.5	12.80				6.47		
	26	3.25	8.00				4.04		

The concentration of MAP fusion antigen on protein particles was calculated by densitometry analysis on purified PhaC-MAP fusion protein particles separated on SDS-PAGE (**Figure S1**). Bovine serum albumin was used as a standard to quantify amount of MAP fusion antigen.

### Vaccination

Thirty-two calves were divided randomly into 4 vaccine groups of 8 animals as shown in **Table 2**. Calves in groups 1, 3 and 4 were vaccinated subcutaneously with 2 mL vaccine in the anterior region of the neck (week 0). Animals were re-vaccinated with the same vaccine 3 weeks after the first vaccination. Calves in group 2 were vaccinated in the same manner as the other groups, but only once with 1 mL Silirum^®^ vaccine.

**Table 2:** Vaccine groups.

Groups	Antigens	Adjuvant
1	PBS	Emulsigen-D
2	Silirum^®^	–
3	Recombinant MAP fusion protein	Emulsigen-D
4	Protein particle displaying MAP fusion protein	Emulsigen-D

Blood samples were collected by jugular venipuncture using blood tubes with no anti-coagulant and heparinized blood tubes (Vacutainer, Becton Dickinson, NZ) before vaccination (week 0) and after vaccination at weeks 3, 6, 9, and 12 to measure antibody titers. For serology, blood was centrifuged at 2,500 × g for 10 min at room temperature and serum was aspirated and stored at –20°C. Heparinized blood samples were used to measure IFN-γ, IL-17A and gene expression in antigen-stimulated leukocytes.

### Antibody ELISA

Serum IgG antibody responses to PPDA and rMAP fusion protein (referred to as recombinant antigen (RA) when used to measure immune responses) were measured by ELISA using a previously described method with some modifications (27). Briefly, Microlon high-binding capacity 96 well ELISA plates (Greiner Bio-One, Germany) were coated overnight at 4°C with 50 µL/well of PPDA or rMAP fusion protein (4 µg/mL) in 50 mM sodium carbonate buffer, pH 9.6. The following day, the plates were washed with PBS + Tween-20 (0.5%) (PBST) and blocked for 1 h at room temperature with 100 µL/well of blocking buffer (PBS containing 1% (w/v) casein). After incubation, the plates were washed with PBST, and 2-fold serial dilutions of sera (range 1:200 – 1:204,800 diluted in blocking buffer) were added (50 µL/well). The pre-vaccination (week 0) and post-vaccination sera (week 3, 6, 9, and 12) of an animal were tested on the same plate. The plates were incubated for 1 h at room temperature, washed with PBST, then incubated for 1 h at room temperature with HRP-conjugated donkey anti-bovine IgG (BioRad, CA, USA) diluted at 1:6,000 in blocking buffer (50 µL/well). Following washing with PBST, 50 µL/well of 3,3′,5,5′-Tetramethylbenzidine (TMB) substrate (BD Biosciences) was added, and the plates incubated for 20 min at room temperature in the dark. The reactions were stopped with the addition of 50 µL/well of 0.5 M H_2_SO_4_ and the absorbance read at 450 nm using a microplate reader (VERSAmax, Molecular Devices). For each animal, the antibody titer of each post-vaccination serum was calculated from the reciprocal of the highest dilution showing an OD_450_ value greater than the OD_450_ value of a 1:200 dilution of pre-vaccination serum.

### IFN-γ and IL-17 assays

Heparinized blood samples were obtained from the calves and within 6 h of collection, aliquots (1 mL) were dispersed into wells of a 48-well plate and either PBS (negative control), pokeweed mitogen (positive control, 2.5 µg/mL final concentration), PPDA (24 μg/mL final concentration; Prionics, Schlieren-Zurich, Switzerland), or RA (10 µg/mL final concentration) was added for IFN-γ and IL-17A whole blood assays. After incubation at 37°C for 24 h, the plasma supernatants were harvested (400 × g for 10 min). IFN-γ levels were measured using a sandwich ELISA kit (Prionics, Thermo Fisher Scientific) and bovine IFN-γ standard (Kingfisher Biotech, St. Paul, USA) was titrated to calculate the concentration of IFN-γ (pg/mL) in each sample and results were expressed using the standard curve.

An ELISA for bovine-specific IL-17A was developed and optimized in-house using capture, detection antibodies and recombinant bovine IL-17A as standards (Kingfisher Biotech, MN, USA) according to the manufacturer’s instructions. The optimized conditions were used to measure IL-17A levels in plasmas from antigen-stimulated whole blood of the animals prior to vaccination (week 0) and at weeks 3, 6, 9, and 12 post-vaccination. Briefly, MaxiSorp high protein-binding capacity 96 well ELISA plates (Nunc™) were coated overnight at room temperature with 50 µL/well of capture antibody (2 µg/mL protein) in PBS. The plates were washed with PBST and blocked for 1 h with 100 µL/well of blocking buffer (PBS containing 4% (w/v) BSA) at 37°C with shaking. Following blocking, the plates were washed again with PBST. Bovine IL-17A standards and undiluted plasma samples (50 µL/well) were added to the plates and the plates were incubated for 1 h at 37°C. Following the incubation, the plates were washed with PBST and incubated for 1 h at 37°C with biotin-conjugated detection antibody (Kingfisher Biotech, MN, USA) diluted at 1:4,000 in blocking buffer (50 µL/well). After incubation, the plates were washed with PBST, and then incubated for 30 min at 37°C with streptavidin-HRP (Kingfisher Biotech, MN, USA) diluted at 1:500 in blocking buffer (50 µL/well). After the incubation, the plates were washed with PBST, and 50 µL/well of TMB substrate (BD Biosciences) was added, and the plates were incubated 20 min at room temperature in the dark. The reactions were stopped by the addition of 50 µL/well of 0.5 M H_2_SO_4_ and absorbance read at 450 nm using a microplate reader (VERSAmax, Molecular Devices). The concentration of IL-17A (pg/mL) for each sample was calculated from the standard curve.

### Measurement of gene expression in antigen-stimulated leukocytes

Leukocytes were prepared from heparinized blood samples using a method previously described with some modifications (31). Briefly, 3 mL of blood was transferred into a 50 mL falcon tube and 13.5 mL of chilled water was added (4.5 mL/ml of blood) to lyse the red blood cells. The tubes were quickly mixed for 15 sec, and 1.5 mL of 10X DPBS (500 µL/mL of blood) (Thermo Fisher Scientific, New Zealand) was added to equilibrate the sample. Subsequently, the cells were centrifuged at 250 × g for 10 min at 4°C, washed with PBS (10 mM, pH-7.3) and re-suspended in 0.5 mL of RPMI-1640 containing 10% fetal bovine serum (Thermo Fisher Scientific, New Zealand). Cell number and viability was measured by trypan blue exclusion method using TC20 cell counter (BioRad). A total of 2x10^6^ cells were added to each well in a U-bottom 96-well tissue culture plate (Nunc™) and stimulated with either media alone (un-stimulated), PPDA (24 μg/mL) or RA (10 µg/mL) at 37°C for 24 h. After incubation, plates were centrifuged in a swing out rotor at 350 × g for 10 min at room temperature. The supernatant was removed and 150 µL of a commercial lysis buffer for RNA preparation was added (RLT buffer, Qiagen, Hilden, Germany) to the samples and the plates were stored at –80°C until RNA isolation. Total RNA was isolated from the samples using RNeasy kit according to the manufacturer’s instructions (Qiagen, Hilden, Germany).

### nCounter analysis of gene expression

Gene expression analysis was performed using the nCounter Analysis System (Nanostring Technologies Inc., Seattle, WA) as previously described (32). The use of NanoString technology enables RNA expression analysis from either purified RNA or directly from cell lysates without further RNA purification or amplification (33). The method uses molecular barcodes on gene-sequence-specific probes and single molecule imaging to count RNA copies (34). RNA was prepared from the antigen-stimulated leukocytes before vaccination (week 0) and after vaccination (weeks 6, 9 and 12) and analyzed using PlexSet-24 consisting of probes specific to 21 immune response genes and 3 reference genes (**Table 3**).

**Table 3:** List of genes analyzed by nCounter.

Accession Number	Target Gene	Possible function
NM_174093.1:330	*IL1B*	Pro-inflammatory cytokines
NM_173923.2:319	*IL6*	
NM_174356.1:874	*IL12B*	
NM_174445.2:1746	*PTGS2/Cox-2*	
NM_174086.1:502	*IFNG*	Adaptive immunity cytokines
NM_001008412.1:147	*IL17A*	
NM_001098379.1	*IL22*	
NM_001166068.1:961	*TGFB*	
NM_180997.2:217	*IL2*	Anti-inflammatory cytokines
NM_173921.2:335	*IL4*	
NM_174088.1:144	*IL10*	
NM_001076259.1:718	*PTX3*	Innate receptors
NM_174197.2:1497	*TLR2*	
NM_174198.6:2640	*TLR4*	
NM_001105611.2:414	*CD40*	T-cell activation markers
NM_174624.2:608	*CD40LG*	
NM_001038017.2:719	*CD86*	
NM_174297.1:35	*CTLA4*	
NM_001039957.1:1178	*ITGAM*	
NM_001083506.1:320	*PDCD1*	
NM_001163412.1:393	*PDL1*	
NM_001083436.1:1814	*GUSB*	Reference genes
NM_001077866.1:553	*RPL15*	
NM_174814.2:146	*YWHAZ*	

A titration was performed using bovine-specific ProbeSets (**Supplementary Data Sheet**) and a PlexSet-24 titration kit according to the manufacturer’s instructions (NanoString Technologies) to optimize the input RNA concentration of each sample in the final PlexSet-24 analyses (data not shown). As a result, a total of 1.1 µg of purified RNA was used to measure the expression of various immune response genes (**Table 2**) using bovine specific PlexSet-24.

For analysis, background subtraction was performed by subtracting the geometric mean of 8 internal negative controls from each sample. Positive control normalization was performed using the geometric mean of 6 internal positive controls to compute the normalization factor. The normalization factor of all samples was inside the 0.65 to 1.67 range.

The geometric mean of counts of the three reference genes included in the ProbeSet was used for gene normalization. The average of these geometric means across all lanes was used as the reference against which each lane is normalized. A normalization factor was then calculated for each of the lanes based on the geometric mean of counts for the reference genes in each lane relative to the average geometric mean of counts for the reference genes across all lanes. This normalization factor was then used to adjust the counts for each gene target and controls in the associated lane. The normalization factor of all samples was inside the 0.5 to 21 range.

Fold-change was calculated by dividing normalized RNA counts for each gene of antigen-stimulated blood leukocytes over media alone stimulated cells at weeks 0, 6, 9, and 12. Ratios were calculated for each gene at weeks 6, 9, and 12 by dividing fold-change (antigen stimulation/media alone) values at weeks 6, 9, and 12 over fold-change expression at week 0. The data were log2 transformed prior to statistical analysis.

### Intradermal tuberculin test

A comparative cervical tuberculin intradermal test was conducted at week 12. For this test, the cattle were inoculated intradermally with 0.1 mL volumes containing either 2,500 IU of PPDA or 5,000 IU of purified protein derivative from *Mycobacterium bovis* (PPDB) (AsureQuality, Upper Hutt, New Zealand) at separate sides on the right side of the neck. The skin fold thicknesses were measured with Calipers prior to and 72 h after injection of the PPDs. Positive skin test responses to PPD were defined as increases in skin thickness prior to injection and 3 days later of ≥ 2 mm and ≥ 4 mm and the differential increase, PPDB-PPDA of ≥ 2 mm and ≥ 4 mm.

### Statistical analysis

The statistical analysis of fold-change of antibodies, cytokine responses and gene expression values was performed using R software version 4.1.1 (35). For each gene, permutation ANOVAs as implemented in the lmPerm R package version 2.1.0 (36) were used to evaluate the significance of timepoint and vaccine. *Post-hoc* testing and calculation of predicted means and 95% confidence intervals were calculated using the predictmeans R package version 1.0.6 (37). P-values < 0.05 were considered statistically significant. The “pca” function within the mixOmics R package version 6.12.1 (38) was used to perform PCA on the gene expression values. The mixomics R package was also used to perform canonical correlation analysis with the shrinkage method to account for a large amount of co-correlation between the gene expression and immunology data. The canonical correlation analysis results, along with Pearson correlations were displayed in a network plot were prepared using Cytoscape version 3.8.2 (39).

## Results

### Recombinant MAP fusion protein induces long lasting antibody responses

**Figure 1:**
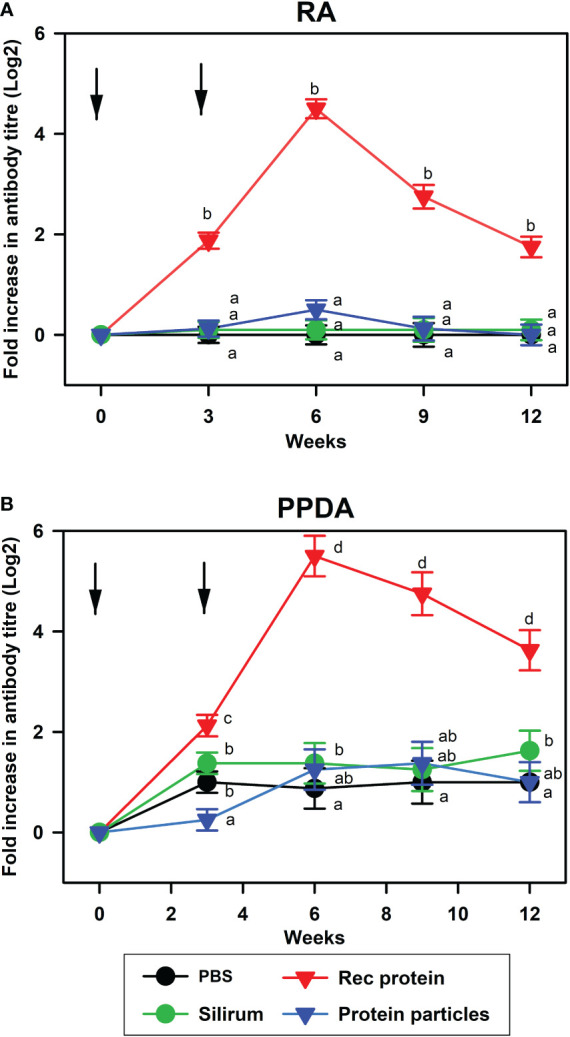
Antibody responses to recombinant antigen and PPDA in different vaccine groups. Mean serum antibody responses to **(A)**, recombinant antigen (RA); and **(B)**, PPDA in calves vaccinated with PBS, Silirum^®^, rMAP fusion protein (Rec protein) and protein particles displaying MAP fusion protein (Protein particles) vaccines at weeks 0 and 3. Timing of vaccinations is indicated by arrows. Antibody titers were measured in sera before vaccination and after vaccination at weeks 3, 6, 9, and 12 using ELISA. Significance differences (P < 0.05) in antibody responses between different vaccine groups are indicated by different letters, while the same letter indicates no significant differences.

Serum antibody responses to RA and PPDA were measured in the vaccinated animals. Antibody levels to RA and PPDA were significantly higher in the animals vaccinated with rMAP fusion protein at 3, 6, 9 and 12 weeks compared to animals vaccinated with PBS, Silirum^®^ or the protein particles (P < 0.05, **Figure 1**). The strong antibody responses induced by the rMAP fusion protein vaccine were long-lasting with peak responses at week 6 and antibody responses still higher than the other groups at week 12 (P <0.05). In comparison, calves vaccinated with the protein particles or given Silirum^®^ produced no significant antibody responses to both RA and PPDA.

### Recombinant MAP fusion protein induces cell-mediated immune responses

**Figure 2:**
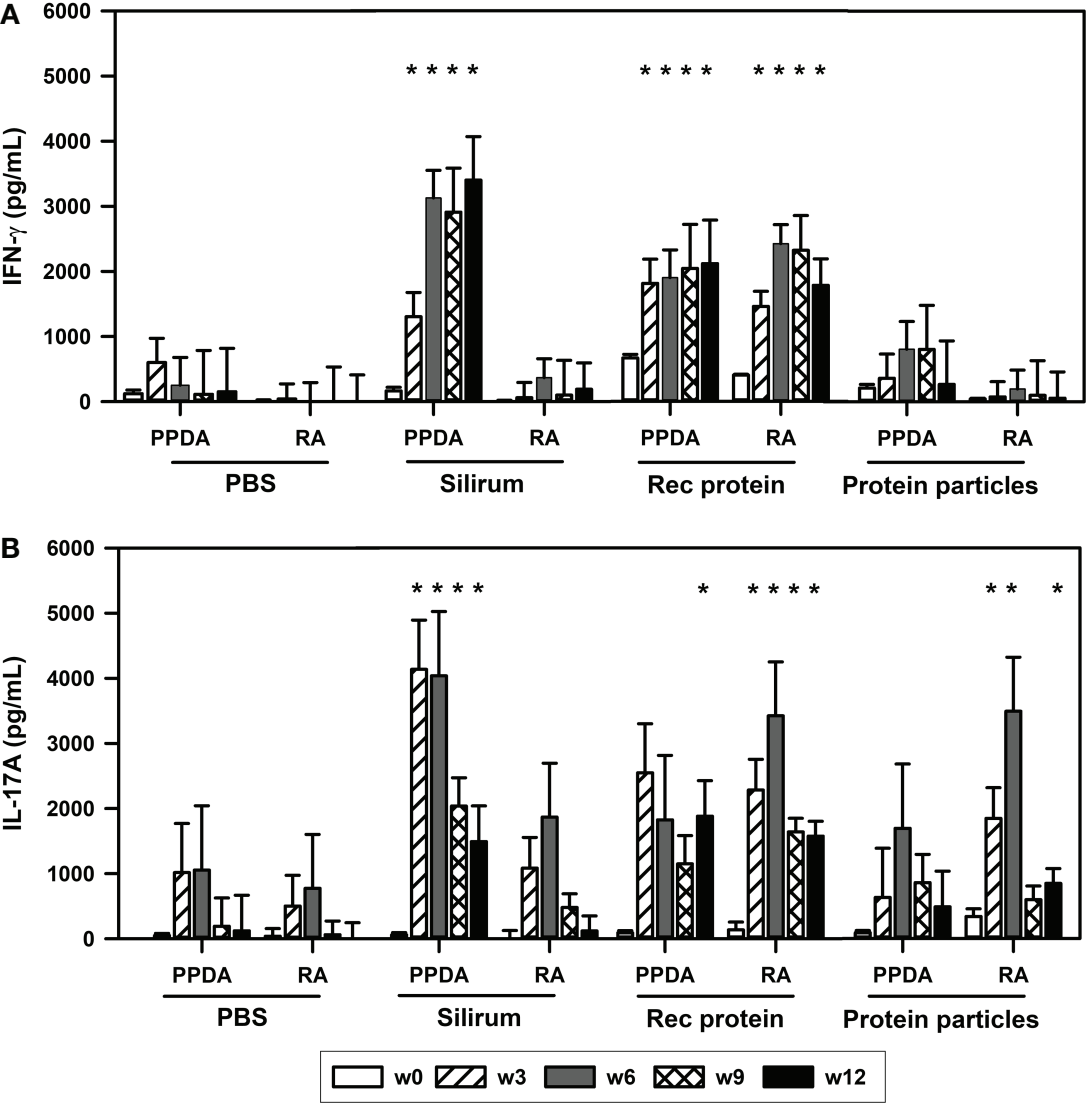
Antigen-specific cell-mediated immune responses in the vaccinated animals. Mean (+ SE) **(A)**, IFN-γ and **(B)**, IL-17A responses in the animals vaccinated with PBS; Silirum^®^; recombinant MAP fusion protein (Rec protein); and protein particles displaying MAP fusion protein (Protein particles). Whole blood from the vaccinated calves at weeks 0, 3, 6, 9 and 12 were stimulated *in vitro* with PBS, PPDA, or RA. IFN-γ and IL-17 levels were measured by ELISA. Results for IFN-γ and IL-17A cytokine levels are presented as difference obtained by subtracting values of PBS- from PPDA- or RA-induced IFN-γ and IL-17A cytokines. Significance indicated as *, P < 0.05 compared with the respective treatment in the PBS vaccinated animals.

The ability of the vaccines to induce T-cell immune responses was evaluated by measuring antigen-specific IFN-γ and IL-17A responses in the vaccinated animals. Calves vaccinated with rMAP fusion protein vaccine elicited antigen-specific cell-mediated immune responses as indicated by increases in IFN-γ levels in blood stimulated *in vitro* with PPDA or RA (**Figures 2A, B**). These responses were significantly higher compared to the PBS vaccinated animals after vaccination at weeks 3, 6, 9 and 12 (P < 0.05, **Figures 2A, B**). IL-17A cytokine levels were also increased significantly at weeks 3, 6, 9, and 12 after stimulation with RA and only at week 9 with PPDA stimulation in the blood of the rMAP fusion protein vaccinated animals compared to the PBS group. In comparison, the MAP fusion protein particle vaccine induced weaker responses with significant increase in IL-17A levels in response to RA observed at weeks 3, 6 and 12 compared to the PBS group (P < 0.05, **Figure 2B**). There were no differences in IFN-γ levels between the MAP fusion protein particle group and the PBS group. The animals vaccinated with Silirum^®^ vaccine also produced significantly higher levels of IFN-γ and IL-17A at weeks 3-12 in response to PPDA but not to RA compared to the PBS group (P < 0.05, **Figures 2A, B**). The range of IFN-γ levels in the pokeweed mitogen stimulated whole blood cells was 1093 pg/mL to 10767 pg/mL indicating responsiveness of the cells (data not shown).

### Recombinant MAP fusion protein induces expression of key genes of various immune pathways

The ability of the vaccines to stimulate various immune pathways was evaluated in antigen-stimulated leukocytes prepared from blood of the immunized animals. Purified leukocytes were stimulated *in-vitro* with PPDA or RA for 24 h and expression of immune response genes was measured using NanoString.

**Figure 3:**
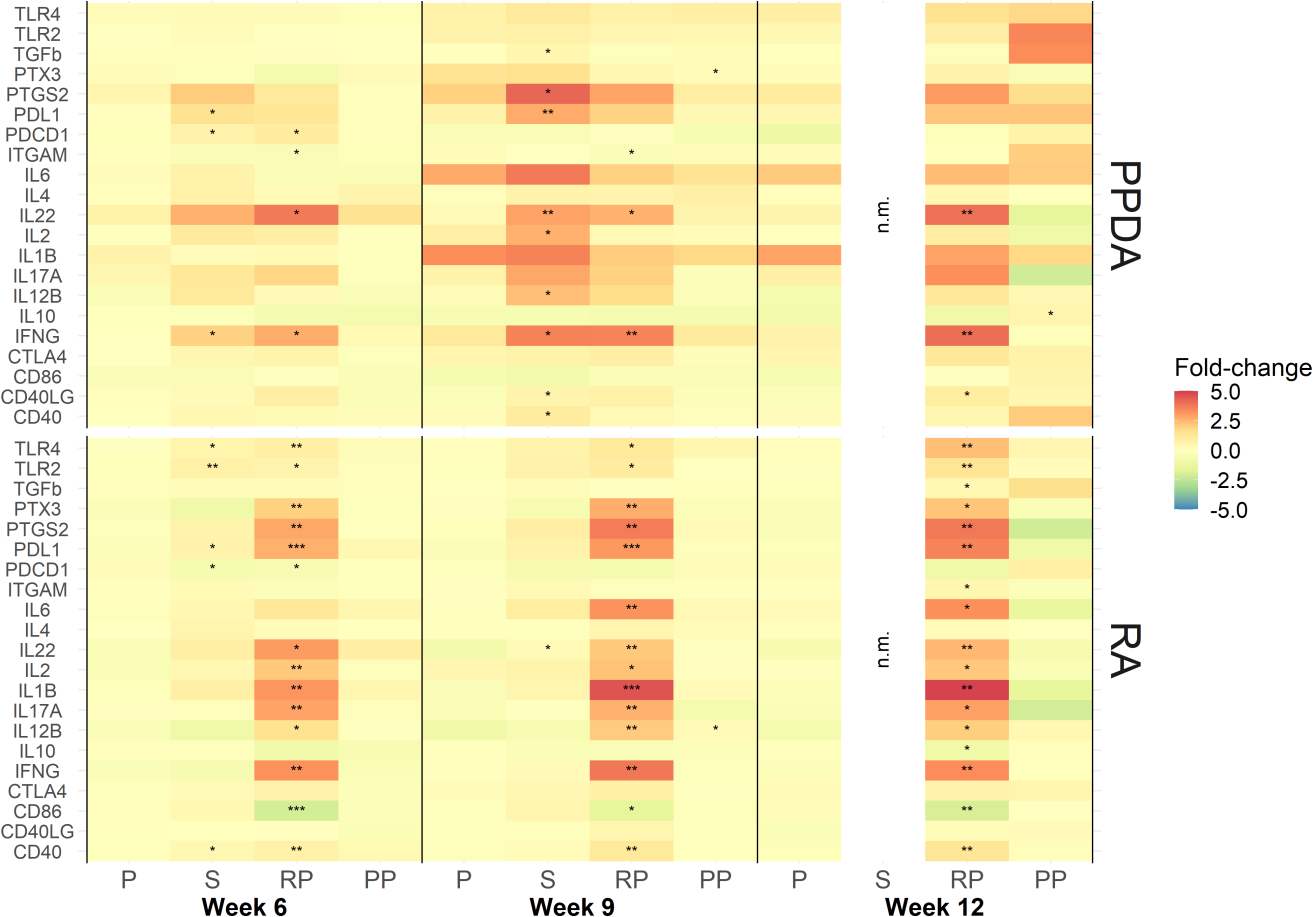
Expression of immune responsive genes in animal vaccinated with PBS **(P)**, Silirum^®^ **(S)**, recombinant MAP protein (RP), and MAP fusion protein particles (PP). Leukocytes were stimulated *in vitro* with either media alone, PPDA or RA at a final concentration of 10 μg/mL for 24 h. Results are presented as ratios of fold-change (antigen stimulation/media alone) for each gene at weeks 6, 9 and 12 over expression at week 0. The data was log2 transformed for statistical analysis and statistical significance was calculated by comparing ratios of antigen stimulation/media alone of all the vaccine groups and week 0 expression of the PBS control group. Statistical significance was calculated compared with the PBS group (* = P < 0.05; ** = P < 0.01; *** = P < 0.001). Note: No responses were measured in Silirum^®^-vaccinated animals at week 12 due to RNA being lost during sample preparation.

Transcription analysis revealed that several immune response genes were significantly upregulated upon re-stimulation of leukocytes from calves vaccinated with rMAP fusion protein compared to animals administered PBS alone. Genes for *TLR4*, *TLR2*, *PTX3*, *PTGS2*, *PDL1*, *IL22*, *IL2*, *IL1B*, *IL17A*, *IL12B*, *IFNG*, *CD40* were upregulated after stimulation with RA at weeks 6, 9 and 12; IL6 at weeks 9 and 12; and *PDCD1* expression at week 6 (**Figure 3**). Expression of *CD86* was downregulated at weeks 6, 9, and 12 and *ITGAM* expression was downregulated at weeks 12. Blood leukocytes stimulated with PPDA showed upregulation of *IL22* and *IFNG* genes at weeks 6, 9 and 12; *ITGAM* at weeks 6 and 9; *PDCD1* at week 6; and *CD40LG* at week 12 (**Figure 3**). Pathway analysis revealed that several of these genes participate in T-cell receptor and IL-17A signaling pathways (**Figures S2**, **S3**).

In the animals vaccinated with the MAP fusion protein particle vaccine, the expression of only a few genes were found to be modulated in antigen-stimulated leukocytes. The expression of *IL12B* and *PTX3* were increased at week 9 in RA and PPDA stimulated leukocytes, respectively, while *IL10* was upregulated at 12 weeks after PPDA stimulation compared to the PBS group.

In the Silirum^®^-vaccinated animals, expression of *PDL1*, *PDCD1*, *IFNG* and *TGFb*, *PTGS2*, *PDL1*, *IL22*, *IL2*, *IL12B*, *IFNG*, *CD40LG*, and *CD40* were upregulated in blood leukocytes stimulated with PPDA at weeks 6 and 9, respectively compared to the PBS group. Leukocytes from these animals stimulated with RA had increases in expression of *TLR4*, *TLR2*, *PDL1*, *PDCD1*, *CD40* and *IL22* genes at weeks 6 and 9, respectively. No samples were analyzed for gene expression at week 12 due to loss of mRNA during sample preparation.

### Correlation between gene expression and cellular immune responses in recombinant MAP vaccinated animals

**Figure 4:**
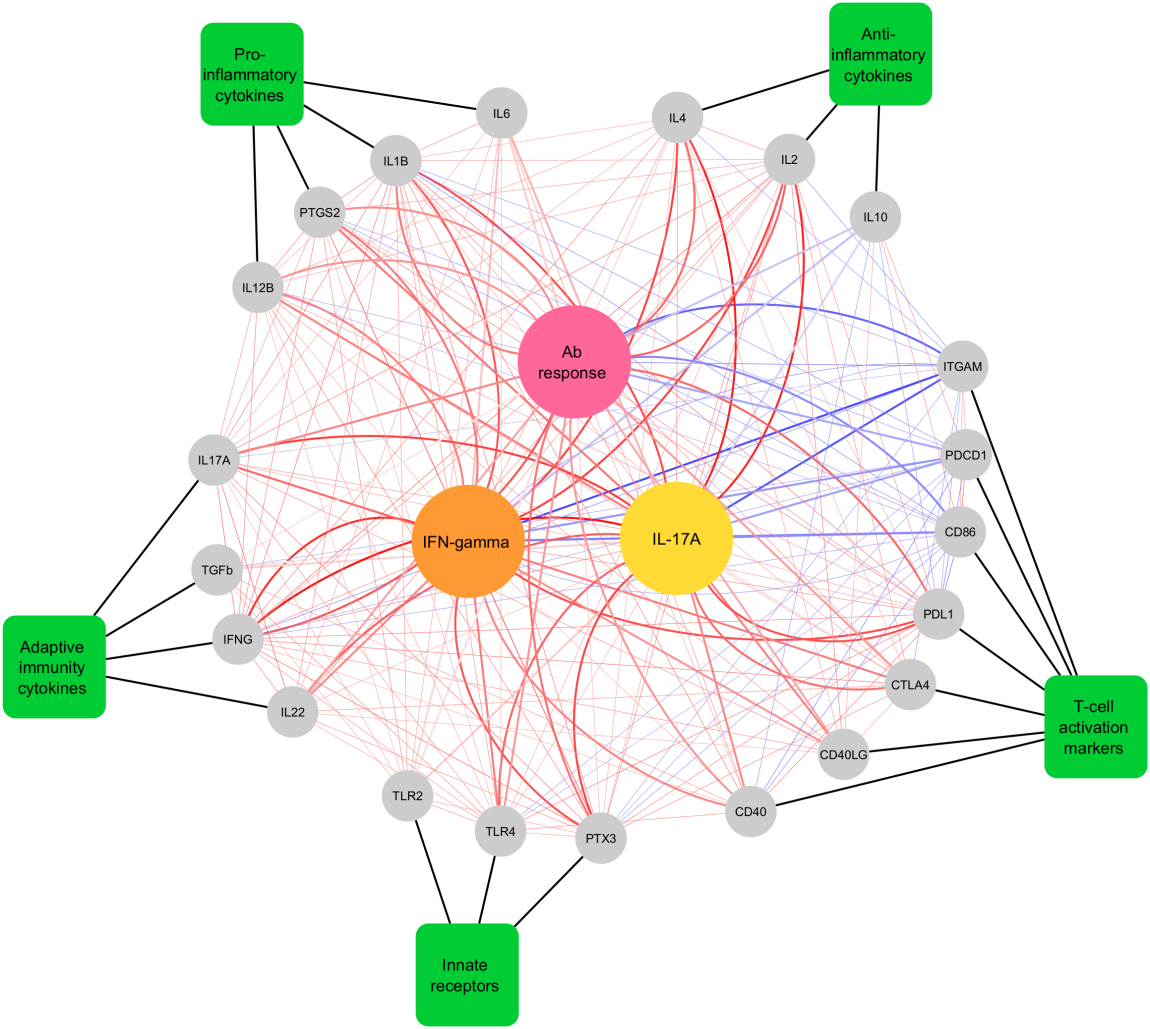
Network plot showing Pearson correlations and canonical correlations between differentially expressed genes, antibody levels and T cell-mediated cytokine responses in cattle vaccinated with rMAP fusion protein at weeks 0, 6, 9 and 12. Genes are displayed as grey circles, gene categories are displayed as green rectangles and the three central circles colored pink, yellow and orange are, antibody, IL-17A (pg/mL), and IFN-γ (pg/mL) responses to RA, respectively. Thick black lines indicate the functional group that the genes belong to. Thick red and blue lines indicate canonical correlations above |0.6| and thin red lines indicate significant (P < 0.05) Pearson correlations between genes. Red lines indicate positive associations and correlations, while blue lines indicate negative associations and correlations. Darker line colors indicate stronger associations and correlations.

We performed network plot analysis to identify correlations between expression of 21 genes in RA-stimulated leukocytes and antigen-specific immune responses (antibody, IFN-γ and IL-17A cytokines) in cattle vaccinated with rMAP fusion protein at various time points (weeks 0, 6, 9 and 12). The analysis revealed expression of several genes involved in various immune pathways were highly correlated with humoral- and cell-mediated immune responses (Cor > 0.6) (**Figure 4**). For example, expression of pro-inflammatory cytokines and innate immune receptors genes including *IL1B*, *IL12B*, *PTGS2*, *TLR4*, and *PTX3* strongly correlated with antigen-specific cellular responses. In addition, T-cell activation and adaptive immunity cytokines genes including *PDL1*, *CD40*, *IL17A*, *IFNG*, and *IL22* highly correlated with antibody responses, IFN-γ and IL-17A cytokines. A few T-cell activation markers including *ITGAM*, *PDCD1*, and *CD86* were negatively correlated with RA-specific antibody, IFN-γ and IL-17A cytokines. Positive and negative correlations between the genes of various pathways were also observed as indicated by light red and light blue lines, respectively (**Figure 4**).

### Recombinant MAP fusion protein does not compromise intradermal skin tests

**Figure 5:**
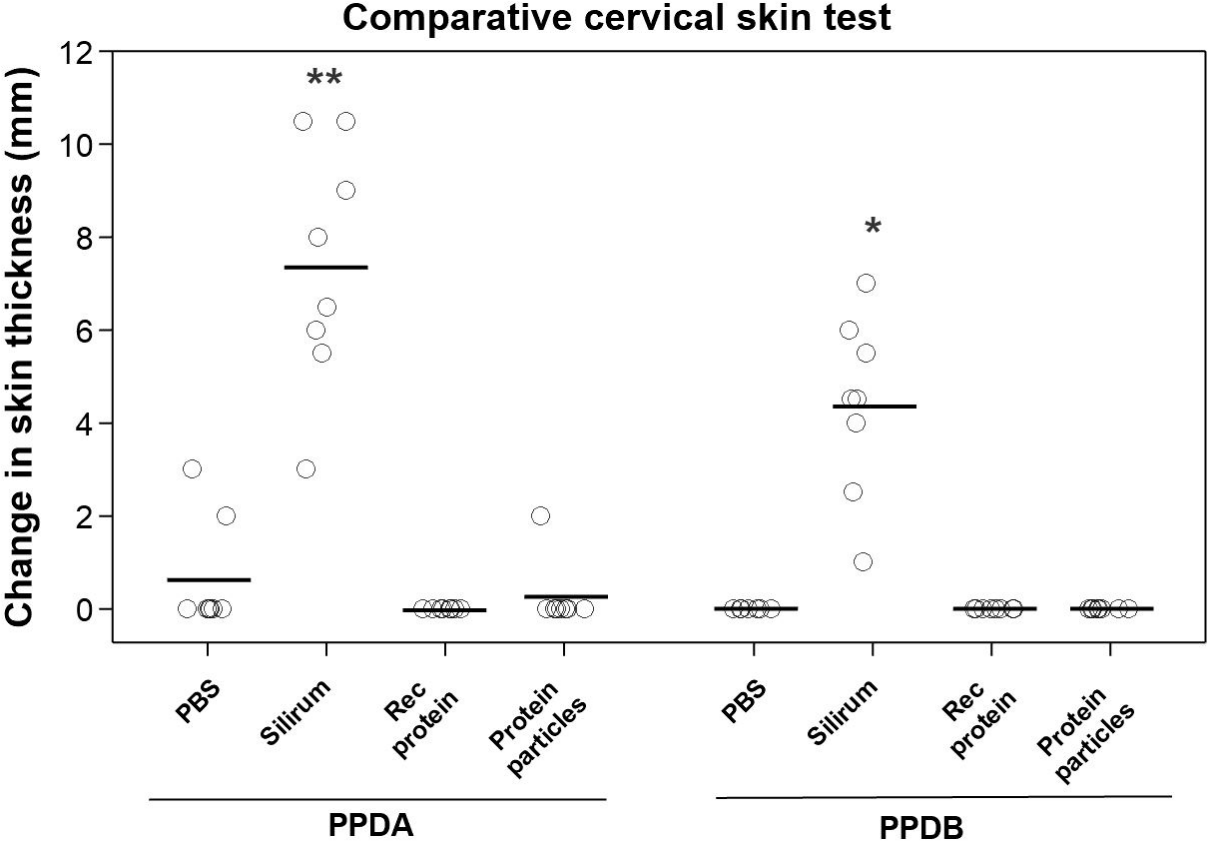
Comparative cervical tuberculin skin test in vaccinated animals. Skin thickness was measured before and at 72 h after injecting 0.1 mL of PPDA or PPDB intradermally at the side of the neck. Mean is represented by ‘─’ for each group. Significance is represented by between different groups. Statistical significance was calculated compared with the PBS control group (* = P < 0.05; ** = P < 0.01).

A comparative cervical skin test was performed in the vaccinated animals to determine if vaccination of calves with the rMAP fusion protein or MAP fusion protein particle vaccines interfered with bovine tuberculosis diagnostic skin tests. Vaccination of calves with either the rMAP fusion protein or MAP fusion protein particle vaccine produced negative responses for both PPDB and PPDB-PPDA as indicated by no significant increase in skin thickness (**Figure 5**). In contrast, seven Silirum^®^-vaccinated calves were positive for PPDB with increase in skin thickness of ≥ 2 mm and six animals had increases of ≥ 4 mm (**Figure 5**). All these calves had a response to PPDA of 3 ≥mm. The differential skin test responses, PPDB-PPDA for the Silirum^®^ group were all negative values (data not shown). Two animals given PBS showed weak reactivity (2- and 3-mm increase in skin thickness) to PPDA, likely reflecting exposure of calves to environment mycobacteria during the time course of the trial.

## Discussion

Johne’s disease (JD) is a severe intestinal disease of ruminants with considerable economic impacts. The currently available commercial vaccine Silirum^®^ can offer some degree of protection to cattle against MAP infection by reducing MAP shedding. But the vaccine interferes with surveillance of bovine tuberculosis by causing reactivity to the tuberculin skin test. In the current study, the immunogenicity of a recombinant MAP (rMAP) fusion protein and a protein particle vaccine displaying the rMAP fusion protein was determined in cattle and compared with immune responses induced by Silirum^®^. Both the rMAP fusion protein and the protein particle vaccines induced antigen-specific T-cell immune responses in cattle, but responses to the particle vaccine were weaker than those generated by the rMAP fusion protein vaccine. These results in cattle did not confirm our previous results in mice, which demonstrated that the protein particle vaccine induced comparable Th1/Th17 cell-mediated immune responses to the rMAP fusion protein vaccine (27). In addition, only the rMAP fusion protein vaccine in the current study induced significant antibody responses to RA and PPDA. In contrast to vaccination with Silirum^®^, vaccination with rMAP fusion protein and protein particles did not compromise the bovine tuberculosis skin test.

The classical Th1 cell-mediated cytokine IFN-γ was significantly upregulated (both at the protein and mRNA levels) in blood cultures from the rMAP fusion protein group after stimulation with PPDA and RA. IFN-γ secreted by T cells (CD4+ and γδ) leads to the activation of antimycobacterial pathways and inhibiting intracellular bacterial growth (40–42). In addition, IL-12 is critical for induction of IFN-γ mediated Th1 protective immune responses (43, 44). In the current study, expression of genes of both these cytokines (*IL12* and *IFNG*) was also upregulated in the rMAP fusion protein vaccinated animals. While IFN-γ has long been considered as a hallmark cytokine in providing protection against mycobacterial intracellular pathogens (45), its role as a sole cytokine in providing protection against mycobacterial pathogens has been challenged (46, 47). Recent evidence indicates that Th17-mediated immune responses also contribute to the early inflammatory responses to mycobacterial infection, thus potentially play a crucial role in providing protective immunity against *M. tuberculosis* and MAP (46, 48–50). The current results demonstrated that antigen-specific IL-17A levels (both at the protein and mRNA levels) were significantly upregulated in animals vaccinated with the rMAP fusion protein. These responses were broadly comparable to responses induced by Silirum^®^ vaccine. These data indicate the ability of rMAP fusion protein vaccine to stimulate both Th1- and Th17-mediated immune responses in cattle, which could provide protective immunity against MAP infection in ruminants.

A balance of Th1/Th17 responses is thought to be important in inducing protective immunity against mycobacteria (51–53). Transcription analysis using NanoString nCounter demonstrated that the expression of several Th1/Th17 immune response genes was upregulated in the vaccinated animals. The results demonstrated that key regulators of Th17 immune responses, including *IL1B*, *IL6*, *IL12B*, *IL17A*, *IL22*, and *TGFB* were upregulated in the rMAP fusion protein vaccine group. Cytokines including IL-1β, IL-6 and TGF-β1 are secreted by the macrophages and/or APCs after antigen stimulation (54, 55), resulting in IL-17 and IL-22 production, which then leads to differentiation of naïve T cell to Th17-like cells (56, 57). In addition, IL-1β and IL-6 cytokines are thought to act in a positive feedback loop on the γδ T cells and on differentiating Th17 cells (57). In our study, increased mRNA levels of *IL1B,IL22* (week 6, 9 and 12) and *IL6* (week 9 and 12) along with increased expression of IL-17A (both protein and mRNA) were observed in the rMAP fusion protein vaccinated animals. Although specific cell types were not characterized in the current study, increased expression of these cytokines as well as *CD40*, a co-stimulatory protein expressed on dendritic cells (DCs) with an essential role in APC activation and Th17 cells differentiation (27, 58, 59), clearly suggest that the rMAP fusion protein vaccine induced Th17 cell-mediated responses in the vaccinated animals. In addition, the expression of IFN-γ (both protein and mRNA) was increased in the rMAP fusion protein vaccinated animals. IFN-γ along with other Th1 cytokines such as IL-1β, IL-2, IL-12 and TNF-α are thought to orchestrate complex immune responses during mycobacterial infection to mount Th1/Th17 balanced immune responses (52, 53). Significantly high levels of mRNA of these cytokines were produced in the rMAP fusion protein vaccinated animals upon stimulation with RA, indicating that balanced Th1/Th17 responses were induced in these animals.

The underlying molecular mechanisms of the host-pathogen interactions are complex in mycobacterial infection. Studies indicate that autophagy, a cell-autonomous host defense against intracellular pathogen, is one of the mechanisms in the host defense against intracellular mycobacterial infection (60, 61). Immune cells including monocytes, macrophages, and DCs recognize mycobacterial molecules *via* TLRs and NLRs to produce cytokines such as IL-1β, IL-12p70, and TNF-α (62–65) and are important regulators of autophagy-mediated host defense against mycobacterial pathogens (66, 67). Therefore, it has been emphasized that vaccines activating autophagy in immune cells will elicit robust innate as well as adaptive immune responses to effectively clear intracellular mycobacterial infection (68–70). There was increased mRNA expression of some of the cytokine genes that participate in autophagy including *IL1B*, *IL6* and *IL12B* in both RA and PPDA re-stimulated leukocytes from both the rMAP fusion protein and Silirum^®^ vaccinated animals. These findings provide some indirect evidence for activation of the autophagy pathway, which could be TLR-dependent as indicated by the increased expression of *TLR2* and *TLR4* in the vaccinated animals. TLR-mediated stimulation and maturation of APCs is essential for T-cell expansion (71) and their role in bridging innate and adaptive immune responses in mycobacterial infection is very well documented (72–74). Studies have demonstrated that mycobacterial antigens activate immune cells including APCs and DCs in TLR2- (63, 75–77) and TLR4-dependent manner (78) to elicit strong Th1 immune responses. Increased expression of *TLR2* and *TLR4* was observed in the animals vaccinated with the rMAP fusion protein and the MAP fusion protein particle vaccine, suggesting potential activation of TLR-signaling in these animals. Further studies will be needed to investigate if the rMAP fusion protein vaccine can provide protective immunity against MAP infection in ruminants.

Additionally, mycobacterial intracellular pathogens can manipulate cell death pathways in infected macrophages, which is another virulence mechanisms of mycobacterial defense against the host (79). Programmed death-1 (*PD-1*) receptor and its ligand, programmed death-ligand 1 (*PDL1*), which is expressed on DCs and APCs, play crucial roles in T cell receptor signaling (80, 81). However, the role of PD-1/PDL1 mechanism in mycobacterial immunity is controversial (82–84). Accumulating evidence suggests that *PDL1* expression on APCs increases during mycobacterial infection (85, 86), leading to the induction of regulatory T cell (Treg) responses (87). Studies on human DCs have demonstrated infection-induced *PDL1* was essential for the expansion of Treg (88, 89). Conversely, *PDL1* deficient mice were increasingly sensitive to tuberculosis infection (83, 90). In the current study, the expression of *PDL1* was induced in the animals vaccinated with rMAP fusion protein (weeks 6, 9 and 12) and Silirum^®^ (weeks 6 and 9), suggesting that these vaccines may be contributing to T cell-mediated immune responses in the vaccinated animals. This observation is in line with a previous study which demonstrated that Bacillus Calmette-Guérin vaccine can induce *PDL1* expression on APCs (85). In addition, prostaglandin-endoperoxide synthase 2 (*PTGS2*), also known as cyclooxygenase-2 (*COX-2*) was upregulated in the animals vaccinated with rMAP fusion protein and Silirum^®^. Like *PDL1*, *PTGS2* has also been associated with Treg-mediated immune responses during mycobacterial infection (87, 91). Overall, the data indicate that the rMAP fusion protein and Silirum^®^ vaccines can induce T cell-mediated immune responses, possibly by activating Treg responses in the vaccinated animals. Further studies are required to characterize functional immune cells in the vaccinated animals and confirm the ability of the vaccine to generate cell-mediated protective immunity.

The role of humoral responses against intracellular pathogens might be undervalued. Antibody-mediated immune responses induced by vaccination could be essential in controlling MAP infection (42, 92–94). In our study, strong antibody responses were observed in the calves administered with rMAP fusion protein vaccine. The increased expression of *CD40* in these animals, which has been implicated in the generation of high titers of class switched and high affinity antibodies (95, 96), suggest that the rMAP fusion protein vaccine-induced antibody responses could provide protective immunity against MAP infection. Furthermore, the expression of pentraxin (*PTX3*) was also increased in the rMAP fusion protein vaccinated animals. *PTX3*, a soluble pattern recognition receptor, is a key component of the humoral arm of the innate immune system and has been suggested as the ancestor of antibodies and the complement cascade (97). These results concur with previous studies, which demonstrated increased expression of *PTX3* in human and bovine macrophages in response to *M. bovis* and MAP, respectively (63, 98). Moreover, studies have demonstrated increased expression of *PTX3* in TLR4-dependent manner and it exhibits opsonizing activity *via* TLR4 pathway during infection (99, 100). In the present study, increased expression of *TLR4* was also observed in the rMAP fusion protein group. While the role of *PTX3* in mycobacterial infection is unclear, it is possible that TLR-mediated opsonizing activity of *PTX3* could potentially promote phagocytosis of mycobacterial pathogens at early stages of infection by the tissue resident macrophages and thus contribute to the innate immune responses during mycobacterial infection.

Often mice are used as the initial model to measure vaccine efficacy, but the immunological responses observed in mice do not always correlate well with the responses observed in large animals (such as cattle, goats, and sheep) (26, 101). This was evident in our two independent studies conducted in mice and cattle. In the current study, the MAP fusion protein particle induced weaker cellular responses compared to the rMAP fusion protein vaccine in cattle, while the protein particle vaccine induced Th1/Th17 cell-mediated immune responses and reduced MAP burden in mice (27). Various studies have demonstrated that different immune responses can be generated for the same antigen when administered as a soluble antigen compared to delivery as a particulate vaccine (102, 103). Several reasons have been proposed for this, such as exposure of different immunostimulatory epitopes on antigens (104, 105), kinetics of soluble and particulate antigen trafficking into lymph nodes (106), processing and presentation of soluble proteins by different mechanisms compared to particulate antigens (14). Due to these possible differences in immune responses, a sequential approach to testing new vaccines is often adopted with large animals such as domestic livestock animals by firstly performing preliminary trials to evaluate their immune responses to the antigens. In the current study, the observed differences in cellular immune responses to protein particles in cattle compared to mice further supports the necessity of firstly performing immunological studies in the target species before proceeding to conduct large, long-duration and expensive MAP vaccine efficacy studies. Therefore, the current study focused on evaluating and reporting the immunogenicity of MAP antigens in two different forms (soluble and protein particles) by determining their ability to induce cellular immune responses in calves.

An important consideration in developing improved vaccines against MAP is the requirement to not interfere with the current on-farm bovine tuberculosis surveillance programme and allow differentiation of infected and vaccinated animals (DIVA). Animals vaccinated with Silirum^®^ vaccine which contains an attenuated MAP strain generated immune responses against both cellular and secreted proteins of MAP, resulting in animals being susceptible to cross-reacting with mycobacterial antigens in PPDA and PPDB. This was evident as the Silirum^®^ vaccinated animals produced positive responses to PPDB. Vaccination with the rMAP fusion protein or protein particles did not induce positive responses to PPDB or to the differential, PPDB-PPDA, in the intradermal skin test, indicating the advantage of using subunit vaccines containing defined mycobacterial antigens with the absence of cross-reactivity to tuberculin. The ability of rMAP fusion protein vaccine to induce strong humoral- and cell-mediated immune responses in the vaccinated animals, has the potential to provide protective immunity against MAP infection without interference with the current bovine tuberculosis skin test.

In summary the ability of two sub-unit vaccines, a soluble recombinant fusion protein of four different antigens of MAP and a protein particle vaccine displaying the same antigens was evaluated to induce antibody- and T-cell-mediated immune responses in cattle. The rMAP fusion protein vaccine induced antigen-specific antibodies as well as IFN-γ and IL-17A cytokines, indicating induction of Th2- and Th1/Th17-mediated immune responses in the vaccinated animals without cross-reactivity to the tuberculin skin test. Notably, transcription analysis of the vaccinated animals indicated upregulation of various genes including *TLR4*, *TLR2*, *PTX3*, *PTGS2*, *PDL1*, *IL1B*, *IL2*, *IL6*, *IL12B*, *IL17A*, *IL22*, *IFNG*, *CD40*, *CD86*, which are important regulators of several immune pathways during mycobacterial host-defense such as autophagy, antigen-presentation, manipulation of macrophage-mediated killing, Th17- and Treg-mediated responses. Taken together, these results indicate that the rMAP fusion protein vaccine induces strong humoral- and cell-mediated immune responses, which could protect cattle against MAP infection without compromising the diagnosis of bovine tuberculosis using the current skin test. These findings provide impetus to evaluate the efficacy of the vaccine in calves experimentally or naturally infected with MAP.

## Data availability statement

The datasets presented in this study can be found in online repositories. The names of the repository/repositories and accession number(s) can be found below: GEO Accession viewer (nih.gov), GSE221544, samples GSM6883433 to GSM6883816.

## Ethics statement

All animal experiments were approved by the Grasslands Animal Ethics Committee, AgResearch and conducted in compliance with the Animal Welfare Act 1999 (the Act) and the Animal Welfare (Records and Statistics) Regulations 1999.

## Author contributions

SG, BB, and DW designed the study. BR selected the antigens for the study. SG performed all experimental work with input from TW, SG, and BB contributed to animal experimental design. AH helped in the Nanostring data analysis. PM performed statistical analysis. SG prepared the manuscript with input from BB, AH, BR, and DW. All authors contributed to the article and approved the submitted version.
