# Supplementary material for: Mycobacterium avium subsp. paratuberculosis antigens induce cellular immune responses in cattle without causing reactivity to tuberculin in the tuberculosis skin test
Source: Front Immunol. 2023 Jan 18;13:1087015. doi: 10.3389/fimmu.2022.1087015 (PMC9889921; doi:10.3389/fimmu.2022.1087015)
Supplement: Supplementary file 1 [file DataSheet_1.docx]

Supplementary Material

# Supplementary Figures

**1 2 3 4 5 6 7 8 9 10**


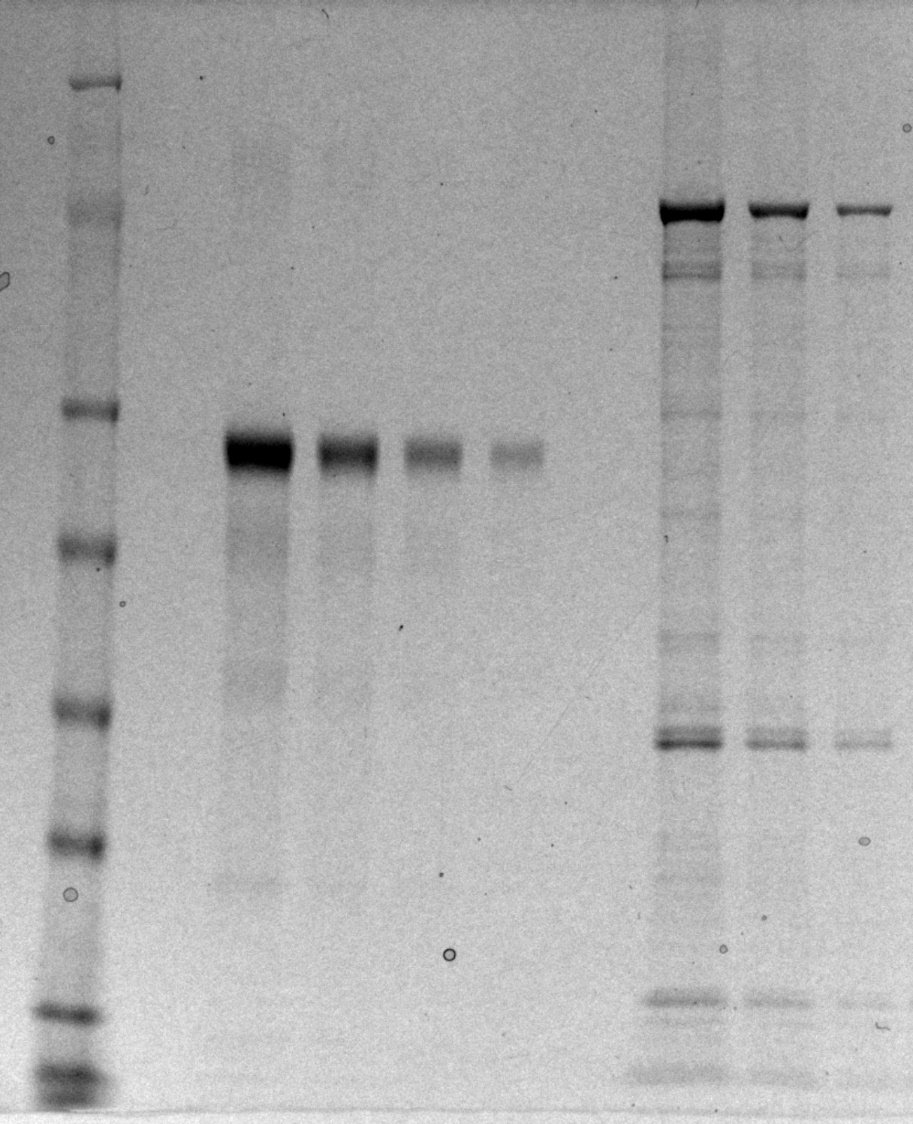


**191**

**97**

**64**

**51**

**39**

**28**

**19**

**14**

**6**

**kDa**

**Supplementary Fig. 1 Densitometry analysis of PhaC-MAP fusion protein particles to calculate concentration of MAP fusion antigen on the particles using bovine serum albumin (BSA) as a standard.** Lane 1, See Blue plus molecular weight marker (kDa); lane 2, empty; lane 3, BSA (500 ng); lane 4, BSA (250 ng); lane 5, BSA (125 ng); lane 6, BSA (62.5 ng); lane 7, empty; lane 8, PhaC-MAP fusion protein particle (15 µg); lane 9, PhaC-MAP fusion protein particle (7.5 µg); lane 10, PhaC-MAP fusion protein particle (3.25 µg).

**Supplementary Fig. 2** **Schematic diagram showing T-cell signaling pathways and interactions of various genes between antigen-presenting cells and T cells, T cells and target cells, Helper T cells and B cells.** Yellow, sky-blue and grey color represent up-regulation, down-regulation and no change in expression of genes, respectively in recombinant antigen stimulated leukocytes from rMAP fusion protein vaccinated animals compared to PBS group (modified from “KEGG map04514 Cell adhesion molecules”; Smith-Garvin et al., 2009).

**Supplementary Fig. 3 Schematic diagram showing IL-17 signaling pathways in various cell types with intermediate transcription factors, cytokines and chemokines.** Yellow, sky-blue and grey color represent up-regulation, down-regulation and no change in expression of genes, respectively in recombinant antigen stimulated leukocytes from rMAP fusion protein vaccinated animals compared to PBS group (modified from “KEGG map04657 IL-17 signaling pathway”; Smith-Garvin et al., 2009).
